# Parental Age and Lifespan Influence Offspring Recruitment: A Long-Term Study in a Seabird

**DOI:** 10.1371/journal.pone.0027245

**Published:** 2011-11-08

**Authors:** Roxana Torres, Hugh Drummond, Alberto Velando

**Affiliations:** 1 Departamento de Ecología Evolutiva, Instituto de Ecología, UNAM, México DF, México; 2 Departamento de Ecoloxía e Bioloxía Animal, Universidade de Vigo, Vigo, Spain; Dalhousie University, Canada

## Abstract

Recent studies of wild populations provide compelling evidence that survival and reproduction decrease with age because of senescence, a decline in functional capacities at old ages. However, in the wild, little is known about effects of parental senescence on offspring quality. We used data from a 21-year study to examine the role of parental age on offspring probability of recruitment in a long-lived bird, the blue-footed booby (*Sula nebouxii*). Offspring probability of recruiting into the breeding population varied over the life of parents and effects age were similar in mothers and fathers. Offspring recruitment was high when parents were roughly 6–12 years old and low before and after then. Effects of parental age on offspring recruitment varied with lifespan (parental age at last reproduction) and previous breeding experience. Offspring recruitment from young and old parents with long reproductive lifespans was greater than that of offspring from parents with short lifespans at young and old ages. For parents with little previous breeding experience recruitment of offspring decreased with their hatch date, but experienced parents were no similarly affected. We found evidence of terminal effects on offspring recruitment in young parents but not in older parents, suggesting that senescence is more likely a gradual process of deterioration than a process of terminal illness. Failure to recruit probably reflects mortality during the first years after independence but also during the fledgling transition to full independence. Our results show effects of parental age and quality on offspring viability in a long-lived wild vertebrate and support the idea that wild populations are composed of individuals of different quality, and that this individual heterogeneity can influence the dynamics of age-structured populations.

## Introduction

Parents can influence offspring phenotype through genetic and non-genetic effects, the latter resulting from factors other than simple nuclear DNA [1]. Understanding evolution by natural selection requires understanding both genetic and environmental influences of parents on offspring fitness [2], [3]. In iteroparous animals reproductive performance generally varies with age [4], [5], suggesting age-dependent parental effects. These effects can affect variation in the force of natural selection across the lifespan whenever different age-classes contribute differentially to the gene pool [6]–[8].

In natural populations, effects of parental age on the number of offspring reared to independence are well documented [9]–[12]. Typically, young and older parents produce fewer offspring than middle-age parents. However, determining only the number of offspring produced could be insufficient to estimate the magnitude of parental age effects if, for example, offspring quality (i.e. offspring survival and reproduction after independence) is also affected by parental age [13]. Some studies in the laboratory suggest that parental age at conception can influence offspring survival after independence and life history trajectory [14], [15]. For example, in the fruit fly *Drosophila melanogaster*, parental age, particularly the age of the mother, influences offspring longevity and the shape of the curve of age-specific mortality: older mothers generally produce shorter-lived offspring, although the exact effect of maternal age on offspring longevity depends on initial genetic heterogeneity among parents [16], [17]. However, the generality and overall importance to other systems of parental age effects demonstrated in laboratory organisms has been questioned [18], [19].

Studies of parental age effects on offspring quality in wild populations have only recently been carried out and results are mixed. In the red squirrel, *Tamiasciusus hudsonicus*, offspring survival from weaning to one year of age declined with increasing mother age [20], and in the great tit, *Parus major*, proportion of young that recruited to the breeding population decreased with female age [21]. Furthermore, great tit females hatched from older mothers displayed a stronger rate of reproductive senescence in the number of hatchlings and recruits produced than females hatched from younger mothers; nonetheless, reproductive lifespan and lifetime reproductive success (total number of recruits produced) were unaffected by maternal age [22]. In contrast, in the red-billed chough, *Pyrrhocorax pyrrhocorax*, parental age did not influence fledglings first-year survival [13]. Hence, in wild vertebrates effects of parental age on offspring performance after independence are yet poorly understood, probably because long-term monitoring of large populations is required.

Intrinsic parental factors may influence offspring during early development or later in life, such as parental germline defects [23]–[24] or egg maternal effects [17], [25]. In birds, age affects germline quality [25]–[27], with potential consequences for offspring development and survival [28], [29]. Old parents may also provide less or poorer parental care [25]. Furthermore, previous reproductive effort could positively or negatively influence the number and quality of offspring produced. In young animals, previous effort could positively influence breeding performance [30]–[32], because experienced parents frequently have greater skills and better quality mates or territories [33]–[36]. However, previous reproductive effort can increase somatic deterioration, reducing parental performance, particularly of older animals [37].

Individual heterogeneity may strongly impact age-reproductive patterns in animals. Recent studies suggest that populations are composed of individuals of different quality [30], [38]. Thus, population-level effects of parental age might result from selective disappearance (or appearance) of certain phenotypes, rather than within-individual processes, such as senescence [13], [39]–[41]. Selective disappearance might occur, for example, when differential mortality of individuals that invest heavily in reproduction leads to progressive disappearance of good reproducers [40]. Detecting parental age effects may be difficult when there is individual heterogeneity because senescence patterns may vary between individuals of different quality [40]. Lastly, effects of parental age may be confounded or obscured by terminal effects, when individuals close to death are sick or in poor condition (terminal illness[42]–[44]), or increase effort on their last reproductive event (terminal investment [45], [46]).

Here, we used data from a 21-year study to examine effects of parental age on offspring recruitment in the blue-footed booby, *Sula nebouxii*, an iteroparous long-lived tropical seabird with lengthy biparental care (up to six months; [47], [48]). In birds, recruitment has been considered a major component of offspring quality [13], and in the blue-footed booby the probability of recruitment varies considerably [49], [50], accounting for substantial variation in individual fitness. Also, age of male and female boobies influence reproductive success [25], [51], [52], with steady increase in the number of fledglings produced until roughly the tenth year of age, followed by progressive decline [51]–[53]. Development and survival of booby chicks before independence are affected by the amount of incubation, defence and food provided by both parents [54], [55]. In this study we examined the associations between parental age and probability of fledglings' recruitment to address four questions: (1) Does parental age influence the probability of recruitment of offspring? (2) Are parental age effects (if present) due to changes over individual lifetimes (i.e. within-individual heterogeneity) or differences in quality of individual parents (i.e. between-individual effects)? (3) Are parental effects related to previous reproductive effort? 4) Does offspring recruitment differ when parents are in their last reproductive event?

## Materials and Methods

### Ethics statement

The work met the Mexican legal requirements about animal welfare and long-term field work was annually supervised and approved by Dirección General de Vida Silvestre, Secretaría de Gestión para la Protección Ambiental (SEMANART permit numbers 517, 574, 5664, 10470, SGPA/DGVS/01323, SGPA/DGVS/3152, SGPA/DGVS/1543, SGPA/DGVS/0491, SGPA/DGVS/1547, SGPA/DGVS/10832, SGPA/DGVS/01916, SGPA/DGVS/00733, SGPA/DGVS/00357, SGPA/DGVS/00505, SGPA/DGVS/00091).

### Study area

The study was carried out in the breeding colony of blue-footed boobies at Isla Isabel, Nayarit (21°52′N, 105°54′W), off the Pacific coast of Mexico. To evaluate the effects of parental age and previous reproductions (number of previous reproductive attempts) over the lifetime on recruitment of fledglings to the breeding colony, we used longitudinal data on the reproduction and offspring of two cohorts of fledglings (1988 and 1989) in a long-term study.

### Long-term study

In every breeding season the reproductive performance of all breeders in two study areas (20,800 m2 and 6,089 m^2^, roughly 400 m apart) was recorded. In each nest laying, hatching and survival of eggs and chicks were registered every 3 days until most chicks reached age 30 days and thereafter every 6 days until age 70 d (at this age, males and females have reached between 98–100% of their skeletal growth, [49]). Chicks that hatched before the start of monitoring were aged by bill and ulna length [49]. Chicks were individually marked within 3 d of hatching with coloured leg bands, which were replaced by numbered plastic bands at age 7–10 days and by numbered steel bands at age 70 days. The band numbers of breeding birds were recorded and confirmed by independent readings on up to three days. Reproduction was monitored during roughly 5 months of every year, until the end of the colony's fledging period.

Blue-footed boobies are highly philopatric to their natal neighbourhoods (median natal dispersal distance: males, 24.1, females, 28.3 m) and show long term fidelity to their first breeding site. Since they apparently disperse from the study colony only rarely [56], [57], [58], the long-term study protocol allows us to record reproductive histories of nearly all recruits from the study areas. Survival of chicks was recorded until nearly the end of the growing period (70 d) but not until complete independence from parents. Blue-footed booby plumage development is completed at roughly 90 d, when fledglings are able to fly [48]. As in many species of seabirds, fledglings go through a transition period where parents feed them for approximately another 30 to 40 days whereas they develop foraging skills [47]. Hence, failure to recruit to the natal breeding population could occur because offspring died soon after we last registered them at age 70 d, subsequently during the transition to independence or during the first years of independent life. Additionally, some offspring could reach sexual maturity but fail to reproduce or disperse to another colony. We have no measure of how many offspring simply failed to reproduce, but dispersal from our study colony to other breeding colonies is low [49], [56], [57], [58]. During the growth period 52% of hatched chicks died but more than 95% of this mortality occurs before age 30 days (García Cerecedo MA, Saavedra Sordo MT and Drummond H, unpublished data), and fledglings are rarely found dead in the colony. In the year (1989) when we registered survival until plumage was completed only five out of a total of 1000 chicks died at age between 70 and 92 d. Thus, mortality during transition to independence and the first years after independence probably accounts for most of offspring failure to recruit.

### Statistical analyses

The probability of recruitment during the first 6 years of life of fledglings from parents of cohorts 1988 and 1989 was analyzed. In this species, females breed for the first time at age two to eight (3.85±0.08) years and males at age one to ten (4.32±0.09) years, and more than 95% of male and female recruits bred for the first time during the first 6 years of life [49]. Individuals that were not recorded breeding during their first 6 years were assumed to have failed to recruit. In our study, most fledglings analyzed had a single parent from cohorts 1988 and 1989. Nevertheless, 215 out of 1601 fledglings had both parents in either cohort 1988 or cohort 1989. To avoid repetition of data, in these cases only a single parent, selected at random, was included in the analyses. If assortative mating by age occurs in a population, a similar pattern of aging effects in both sexes could arise from senescence in only one sex. However, this does not seem to be the case in the studied population, in a breeding season no correlation between ages of mates was found [53].

The linear and quadratic effects of parental age on fledgling recruitment, including the identity of the parent as a random factor, were analysed using Generalized Linear Mixed Models (GLMMs) with Binomial error distribution, a logit link function (GLIMMIX) [59] and the Satterthwaite approximation for the denominator degrees of freedom [60]. We included parental sex and its interactions with the linear and quadratic effects of parental age to test for differences between maternal age and paternal age on the probability of offspring recruitment. To assess whether decline in the probability of offspring recruitment at older parental ages was significant, General Additive Models (GAMs) were used to determine parental age at onset of senescence (the predicted peak by the GAM). The GAM models were fitted with binomial error distribution, a logit link function, and spline smoothers selected by generalized cross validation (GAM procedure in SAS) [59]. Then, a GLMM (as described above, but excluding the quadratic effects) was adjusted to data from parental age at the onset of senescence (as predicted by the GAM) onward.

To probe the potential mechanisms underlying observed patterns of senescence [39], we performed an additional GLMM as described above but which included as covariates: recruiting age of parent, parental age at last observed reproduction (an estimation of the individual reproductive lifespan), number of previous breeding attempts by parent, fledgling's hatch date (number of days between laying of the egg the fledgling hatched from and the laying of the earliest clutch in the same year) to test for effects of early conditions on offspring recruitment, parental cohort (1988, 1989) and the annual proportion of recruitment from each cohort included in our analyses (1991 to 2003) to control for differences in recruitment due to annual variations. Parental age at last reproduction was analysed up to 2005, i.e. maximum ages 17 and 16 for individuals from cohorts 1988 and 1989, respectively. This is an appropriate estimation of age at last reproduction because roughly only 5% of individuals are still alive at ages 17 [53]. Parental age at last reproduction was included in the model to account for selective disappearance from the breeding population and differences in senescence patterns between parents of different lifespan [39]. To evaluate the potential of terminal effects, we included a two-level variable for parents' last reproductive event (last or not). The identity of the parent was included as random effect. Initially all explanatory variables and their two-way interactions, and the quadratic terms for parental age, parental age at last reproduction and previous breeding attempts were fitted in the model. To test for difference in the effects of maternal and paternal age on offspring recruitment, two and three-way interactions with sex were also included. Nonsignificant terms were dropped sequentially starting with three-way interactions until the minimal adequate model was obtained. Interactions between parental age (linear and quadratic) and parental age at last reproduction tested whether age effects differ between birds of different lifespan while the interactions between parental age and parents' last reproductive event tested the age-specific effect of terminal effects. Statistical significance of random effects was assessed using the restricted likelihood ratio test as the change in −2 log-likelihood of the model with and without the individual effect. This difference is distributed as χ2 [60].

## Results

**Figure 1:**
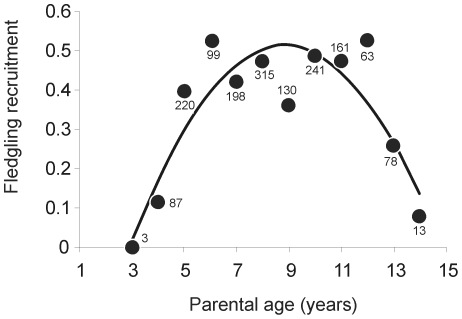
Parental age effects on fledgling recruitment. Circles represent the proportion of fledglings that recruited from each parental age class. Numbers next to circles are sample sizes.

Parent age had a significant quadratic effect on the probability of offspring recruitment (GLMM, parental age, *F*_1,1601_ = 33.47, *P*<0.0001; parental age^2^, *F*_1,1601_ = 31.18, *P*<0.0001; Figure 1), and there was no evidence that maternal and paternal age effects differ (parental age*sex, *F*_1,1598_ = 0.11, *P* = 0.74, parental age^2^*sex, *F*_1,1600_ = 0.02, *P* = 0.89). The age spline in GAM was also significant (*P* = 0.0001), revealing that fledgling recruitment increased steadily up to parent age of five years, followed by a plateau and an abrupt decline after parent age of 12 years. In the analysis of parents of age 12 and older, there was a significant negative linear effect of parent age on recruitment (estimate, −1.20, *F*_1,152_ = 13.81, *P*<0.001).

**Figure 2:**
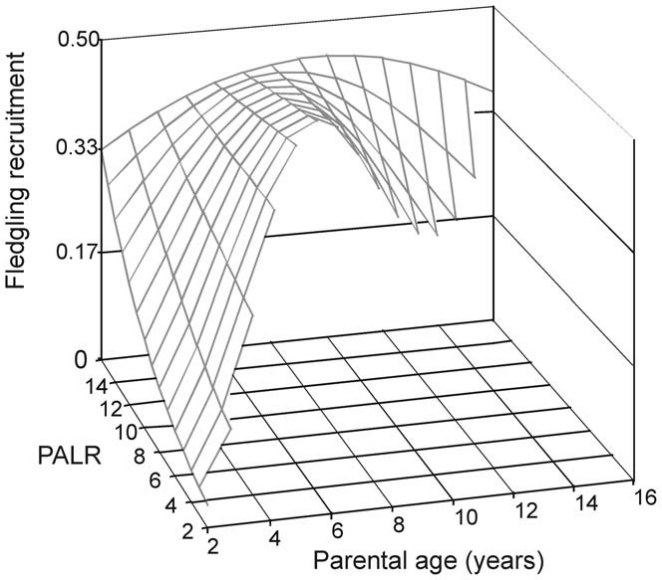
Effect of age of last reproduction (PALR) and age of parents on fledgling recruitment. Estimated surface was calculated from the minimal adequate model reported in table 1.

The additional model to test potential mechanism (see methods) confirmed a quadratic effect of parental age on offspring recruitment (Table 1). In this model, the interaction of parental age^2^ and parental age of last reproduction (parental longevity) on fledgling recruitment was significant (Table 1). Thus, fledglings from long-lived parents had higher recruitment probability and a lesser quadratic effect of parental age compared to fledglings from shorter-lived middle aged parents (Figure 2).

**Table 1:** Effect of parental age and life history traits on fledgling recruitment during the first 6 years of life.

Variable^a^	Estimate (SE)	F^c^	P
Intercept	−8.75 (2.90)		
Parental age (PA)	2.29 (0.80)	8.15	0.0044
PA^2^	−0.167 (0.055)	9.43	0.0022
Parental breeding experience (PBE)	−0.11 (0.06)	3.05	0.081
Parental age of last reproduction (PALR)	0.41 (0.22)	3.71	0.054
Parental last reproduction (PLR)	−1.86 (0.78)	5.63	0.018
Fledgling hatch date (FHD)	−0.026 (0.0053)	23.24	<0.0001
Cohort recruitment	4.90 (0.70)	49.28	<0.0001
PALR*PA	−0.13 (0.59)	5.14	0.023
PALR*PA^2^	−0.0099 (0.038)	6.93	0.010
PLR*PA	0.23 (0.086)	7.56	0.0060
FHD*PBE	0.0033 (0.00096)	11.90	0.0006
Parent identity			<0.0001^b^

Data from 1601 fledglings, from 358 parents that fledged in 1988 and 1989 were analyzed.

a Minimal adequate model. The initial model included parental age (linear and quadratic), sex, patrental cohort, cohort recruitment, fledgling hatch date, recruiting age of parent, parental age at last reproduction, and number of parental previous breeding attempts as fixed variables and parental identity and parental cohort as random factors.

b Statistical significance of random factors was analyzed by restricted likelihood ratio test.

c Degree of freedom, 1,1589.

**Figure 3:**
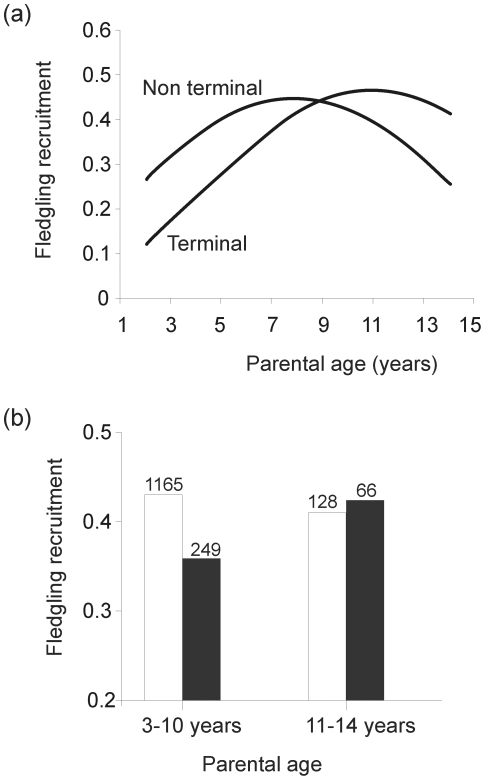
Effect of parental last reproduction (terminal event) on the relationship between parental age and fledgling recruitment. a) Estimated curves calculated from the minimal adequate model reported in table 1. b) Proportion of fledglings that recruited from total fledglings produced (numbers of total fledglings above bars) by age classes from parents in their last reproduction (black bars) or in a non-terminal event (white bars).

**Figure 4:**
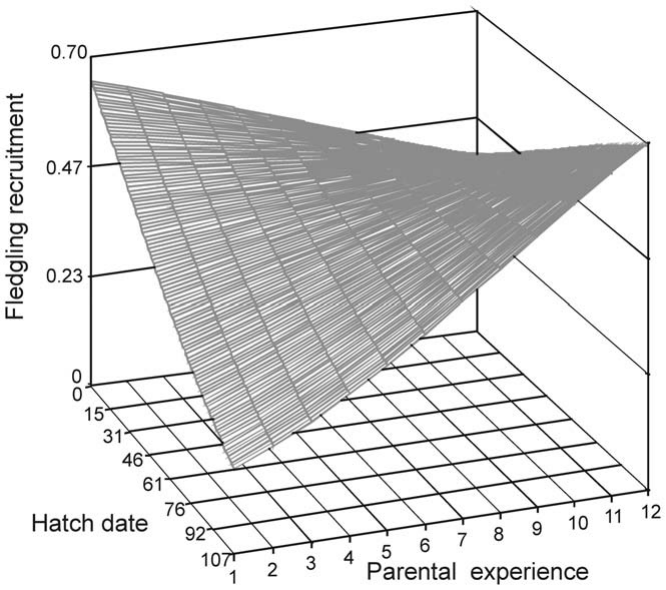
Effect of hatching date and parents' previous breeding experience on fledgling recruitment. Estimated surface was calculated from the minimal adequate model reported in table 1. Hatch date was expressed as the difference in days between the laying date of the egg the fledgling was born from and the laying date of the earliest clutch in the same year.

We also found evidence of terminal effects, especially in offspring of young parents (Parental last reproduction*Parental age, Table 1; Figure 3). Fledglings from young parents (<10 years) in a terminal reproduction had lower recruitment probability compared to fledglings from non-terminal parents (Figure 3b), but this effect was not found in old parents (Figure 3b). Also, the interaction of number of parental previous breeding attempts and fledgling hatch date was significant (Table 1). The probability of fledgling recruitment decreased with fledgling hatch date for parents with low experience, but not for high experienced parents (Figure 4). There was no evidence that maternal and paternal age effects differ (sex and its second- and third-order interactions with main effects, *P*>0.11). Offspring recruitment covaried with cohort recruitment from population (Table 1), but the effects of parental cohort and parent recruiting age were not significant (*P*>0.63).

## Discussion

We found that offspring recruitment probability varies over the life of parents but, interestingly, these effects were modulated by parental life-history, suggesting individual heterogeneity in parental effects. We did not detect any differences between mothers and fathers in the effects of parental age on the probability of offspring recruitment. Overall, fledgling recruitment was highest when either parent was roughly 6 to 12 years old, while younger and older parents produced fledglings less likely to recruit. The decline in recruitment of fledglings produced by senescent parents was confirmed by a posteriori analysis on the data subset after age 12 years, the parental onset of senescence. In the blue-footed booby reproductive success varies with age, and males and females between roughly 8 to 10 years of age produce more fledglings than younger or older parents [25], [51], [53]. Hence, assuming recruits of different aged parents enjoyed similar reproductive success, our long term study suggests that some age classes contribute significantly more to the gene pool than others by producing more fledglings with higher probability of recruiting to the breeding population, thus affecting the strength of selection across parental life.

Effects of parental age on offspring recruitment varied with parental age at last reproduction, an estimation of the parent's reproductive lifespan. Probability of recruitment of offspring from parents of shorter reproductive lifespan was low at young parental ages increasing up to the age of roughly 8 years and then decreasing. In contrast, probability of recruitment of offspring from parents of longer reproductive lifespan was relatively high at young parental ages, increased slightly up to the age of roughly 8 to 9 years, then declined slightly with advancing age. The recruitment enhancement until parental age 8 years may be attributed to the combined effects of individual improvement and selective disappearance of low quality individuals. For parents that differ in their lifespan, reports of age-dependent variation in reproductive traits underlying recruitment have been mixed [11], [21], [38]. Parents' reproductive lifespan influenced offspring birth weight, but not calving date in red deer [11], improved laying date and clutch size in mute swans [38], but did not influence clutch size, hatching success or fledging success in great tits [21]. Similar to what we found, reproductive lifespan of female great tits was positively correlated to annual recruit production, however, contrary to our study, lifespan was not related to senescence in recruitment success [21]. In the blue-footed booby, age at last reproduction of males and females was unrelated to age-dependent variation in laying date, brood size, and breeding success [53]. Nevertheless, we found that parental senescence in offspring recruitment varied with parental lifespan (i.e. between individual heterogeneity). Importantly, the effects of parental age on offspring recruitment were not related to selective disappearance of individuals that invested heavily early in the life. On the contrary, we found that parents with long lifespans produce offspring with higher recruitment probability than those produced by parents with short lifespans. Thus, despite parents of different reproductive lifespans made a similar breeding effort up to fledging [53], parents with long lifespans produced offspring more likely to recruit at younger and older parental ages, compared to parents with short lifespans. Additionally, senescence patterns (onset and slope) in offspring recruitment were more pronounced in parents with intermediate lifespans than parents with long lifespans. Thus, parental quality seems to affect offspring recruitment throughout parental life. Overall, these results suggest that age effects on the probability of offspring recruitment can be attributed to changes within individual parents over their lifetimes (improvement followed by senescence), but largely determined by individual quality (reflected by reproductive lifespan).

In our study, terminal illness, was found in short-lived but not on long-lived parents (i.e. age-dependent), suggesting also heterogeneity in relation to lifespan. Thus, results suggest that for younger birds, terminal illness probably account for their lower breeding success. Fledglings from young parents (<10 years) in a terminal reproduction had lower recruitment compared to fledglings from non-terminal parents, indicating abrupt phenotypic deterioration in the last year of the life. For older parents, the adjusted curves suggest greater investment during a terminal event than in a non-terminal event, but the difference was not significant. Our results suggest that a gradual age-dependent senescence in long-lived individuals, rather than an abrupt decline in performance in their last reproduction. A steep decrease in reproduction in late-life has been found in several bird species [42], [43]. Here, we found that terminal illness may occur in some age classes but not in others, highlighting that terminal effect may be affected by individual quality. Short-lived parents are probably more constrained, due to their physiological condition or their experience/skills, than long-lived parents.

In the blue-footed booby, the probability of recruitment decreases with laying date [49] and old males and females tend to lay late in the season [53]. In birds, recruitment varies among cohorts [61], [62] and within a cohort, early fledglings are often raised in good breeding conditions [63]–[65]; and early incorporation to the breeding population confers advantages throughout life [66]–[68]. Interestingly, in the present study besides the effects of parental age, recruitment decreased with fledgling hatch date for parents with low experience, but not for those with high experience. More experienced parents, those with more than eight breeding events, were able to produce high-quality offspring with high probability of recruiting into the breeding population even late in the season, when environmental conditions are poor. Furthermore, more experienced parents are also parents with longer reproductive lifespans suggesting that individual quality and breeding experience may have additive effects on offspring recruitment. These results highlight how parental effects such as breeding experience can modify chick rearing conditions and overcome the negative effects of late hatching.

We found no effect of parental age at first reproduction, a life history trait that is expected to influence senescence, on the recruitment of offspring from parents of different ages. In the blue-footed booby there is evidence that early onset of a male's first reproduction accelerates, to some extent, reproductive senescence and decreases lifespan [53]. Nevertheless, early onset of reproduction by males and females did not prejudice the recruitment of the offspring they produced at advanced ages, as found in studies of reproductive senescence in the red deer and the red-billed chough [11], [13].

The mechanisms underlying differences in the probability of recruitment of offspring from young and old parents are probably different, but depend on other life history traits and individual heterogeneity. Low recruitment of fledglings from younger parents is probably due to constraint or restraint of inexperienced breeders [33], [69]–[71]. Old parents may also restrain their reproduction in accordance with their individual levels of somatic damage to increase the number of future reproductive events [72]. The fact that variation in recruitment of offspring from younger and older parents was related to individual reproductive lifespan suggests that age-intrinsic factors such as egg quality, rearing capacity [25], and germline deterioration [17], [28], [73]–[75] differ among individuals of different age and quality. In the blue-footed booby older mothers produce smaller eggs and provide lower quality care [25]. Furthermore, older males carry greater loads of premutagenic DNA damages in the sperm [27]. Hence, progeny of blue-footed booby senescent males are probably at higher risk of genetic disorders. Further work will need to investigate the possible physiological and epigenetic effects associated with age and reproductive lifespan and its transmission across generations.

In conclusion, in this long-lived wild vertebrate parental aging appears to affect the viability of offspring. The diverse effects of aging on reproductive performance recently demonstrated in wild populations [10], [40] oblige us to acknowledge the importance of senescence in nature [37], [76], [77]. Our results suggest that senescence is not the result of terminal illness but a gradual process, although short lived parents showed deterioration (as revealed by offspring recruitment) in their last reproductive event. Parental individual heterogeneity influenced recruitment success, with long-lived (i.e. high-quality) breeders performing better than short-lived breeders at younger ages and suffering from lower rates of senescence. Hence, intrinsic-individual factors and probably other life-history traits influence the rate of senescence. Here, we showed that long-lived individuals contributed considerably more to the next generation. Our study highlights that effects of parental age and parental quality are evolutionarily important and should be further incorporated into theoretical models of the evolution of senescence and the dynamics of age-structured populations (see also [78]). Further studies should address which proximate mechanisms are involved in variation of offspring recruitment with parental quality and age.
